# A Theory of Food Exploration with Gender Differences: Childhood Eating Habits and Implicit Food Attitudes

**DOI:** 10.3390/nu16193373

**Published:** 2024-10-03

**Authors:** Omer Horovitz

**Affiliations:** 1 The Physiology & Behavior Laboratory, Tel-Hai Academic College, 9977 North Districts, Qiryat Shemona 1220800, Israel; omerho1@telhai.ac.il; 2 Psychology Department, Tel-Hai Academic College, 9977 North Districts, Qiryat Shemona 1220800, Israel

**Keywords:** nutritional psychology, theory of food, childhood eating habits, implicit attitudes, food choices

## Abstract

Background/Objectives: The growing interest in nutritional psychology has sparked explorations into how eating habits impact one’s emotional, cognitive, and physical health. The Theory of Food (ToF) posits that childhood eating patterns shape food choices in adulthood, influenced by cognitive and associative representations formed early in life. This study explores the relationship between specific childhood eating habits—fruit and snack consumption—and implicit attitudes toward these food groups in adulthood. It also investigates whether this relationship differs by gender. Methods: One hundred and nineteen participants completed a recall questionnaire about their childhood eating habits and an Implicit Association Test (IAT) to assess implicit attitudes toward food groups. The primary hypotheses were that greater fruit consumption in childhood would lead to more positive attitudes toward fruits, and higher snack consumption would result in more favourable attitudes toward snacks. Results: The results did not support the initial hypotheses, indicating no direct relationship between the consumption of fruits or snacks in childhood and implicit attitudes in adulthood. However, further analyses revealed a significant difference in implicit attitudes toward fruits below versus high childhood fruit consumption participation, particularly among women. Conclusions: These findings highlight the complexity of the relationship between childhood eating habits and implicit food attitudes. While no direct associations were found for the overall sample, the significant differences in attitudes based on childhood fruit consumption in women suggest gender-specific patterns. These results emphasize the need for further research to unravel the intricate connections between early eating behaviours and later food attitudes.

## 1. Introduction

In recent years, there has been growing interest in nutritional psychology, particularly in understanding how dietary habits influence mental, emotional, and cognitive states. Numerous studies have highlighted the role of one’s diet in shaping their cognitive, behavioural, and physical health, as well as their mental well-being and social relationships [1,2,3]. These effects are deeply intertwined with the social context of food consumption [4].

Since the development of the Implicit Association Test (IAT) by Greenwald [5], it has become widely accepted that individual behaviour is guided not only by explicit attitudes but also by implicit attitudes—associations that operate below conscious awareness [6]. Implicit attitudes, which influence various behaviours, including nutritional choices, have been explored across multiple domains [7]. According to the Theory of Food (ToF), individuals develop a “food language” during childhood, shaped by the foods they consume and the surrounding social and environmental contexts. This “food language” can have long-lasting effects that persist into adulthood [8,9]. Consequently, childhood dietary patterns may influence implicit attitudes toward food later in life.

This study aims to explore aspects of the ToF by examining the relationship between childhood eating habits and implicit food attitudes in adulthood. Specifically, it investigates whether childhood dietary experiences shape implicit attitudes toward certain food groups in adulthood.

1. Novelty and Contribution of this Study

While numerous studies, particularly in Europe, have utilized longitudinal data to explore the relationship between childhood eating behaviours and dietary patterns in adolescence or early adulthood, many have focused on explicit attitudes or long-term behavioural outcomes [10,11]. These studies have established a positive association between early-life dietary patterns and later eating habits [12,13,14]. However, this study offers a novel contribution by shifting the focus from explicit to implicit attitudes toward food in adulthood, as measured by the Implicit Association Test (IAT) [5]. This study’s integration of the Theory of Food (ToF) provides a fresh perspective on how early dietary experiences may create lasting, unconscious associations with food, influencing implicit adult preferences and behaviours [8,9]. Furthermore, by examining gender differences in implicit food attitudes, this study adds a unique layer to understanding how childhood eating habits affect adult food choices. Using the IAT to explore these implicit attitudes represents a methodological innovation in this field, offering more profound insights into subconscious influences on food behaviours that may not be captured by explicit measures alone.

2. Theory of Food (ToF)

The ToF posits that eating habits and food choices create cognitive associations, similar to how the Theory of Mind (ToM) addresses the understanding of mental states like beliefs and intentions [15]. The ToF suggests that early childhood food experiences form cognitive networks that persist into adulthood, influencing future food preferences and behaviours. Just as children learn about the social world through observation and interaction, they develop associations with food that become part of their cognitive framework as adults.

Research has consistently shown connections between one’s food choices, mood, physical health, and mental well-being [16,17]. Eating patterns are shaped by visible factors such as the cost and taste of food and hidden influences like culture and branding [18]. Tools like the IAT help reveal implicit food preferences that are not always consciously acknowledged. For example, IAT results can predict food choices and show how implicit attitudes influence behaviours differently from explicit attitudes [7]. Additionally, implicit attitudes can steer attention toward taste-related features of food packaging, particularly among those with positive associations toward unhealthy foods [19]. IATs benefit socially sensitive contexts, where implicit attitudes may impact behaviours more than explicit ones [5]. Overall, the IAT has proven to be a reliable tool in predicting dietary choices and revealing the influence of implicit attitudes on consumer behaviours [20,21].

3. Implicit Attitudes

The study of implicit processes—automatic and unconscious judgments that shape behaviours—began in the mid-20th century [22]. The human mind categorizes information naturally, leading to implicit attitudes that often go unrecognized but still influence behaviours [23,24,25,26]. An *attitude* is a psychological tendency expressed through an individual’s positive or negative evaluation of a particular object, encompassing emotions like desire, aversion, and preference [27]. Attitudes are generally categorized into cognitive, emotional, and behavioural components [6,28]. While these components are often conscious, social context can cause certain attitudes to remain implicit [29].

The relationship between implicit and explicit attitudes is complex. While some studies have shown an alignment between the two, particularly in social cognition [29], others suggest they often diverge [30]. Despite this, implicit attitudes have been found to predict spontaneous behaviours, such as maintaining social distance in certain situations [31,32,33]. By measuring explicit and implicit attitudes, researchers gain a deeper understanding of behaviours that social or subconscious factors may influence [34]. IAT tasks are precious in uncovering attitudes that individuals may not be aware of or be willing to report.

4. Gender and Food Choices

Research indicates that gender plays a significant role in food consumption patterns and preferences. Studies have shown that women are generally more likely to adopt healthier eating habits, such as higher fruit and vegetable consumption. At the same time, men may gravitate toward higher-calorie, less-nutritious foods [35,36]. Women are often more concerned with the health implications of their food choices, whereas men may prioritize taste and convenience [37,38]. Additionally, gender differences in socialization and cultural expectations contribute to variations in food preferences, with women frequently facing societal pressures to conform to ideals of health and appearance [36,39]. Understanding the mechanisms behind these gender differences is crucial. Biological factors, such as hormonal influences, may affect one’s appetite and food preferences, while socialization processes shape attitudes and behaviours toward food from an early age. These differences can lead to distinct implicit attitudes toward food that vary by gender, underscoring the importance of considering gender when examining food attitudes and behaviours.

5. The Current Study

This study investigates the relationship between childhood eating habits and implicit food attitudes in adulthood, guided by the Theory of Food (ToF). The ToF suggests that early dietary experiences create lasting cognitive associations with food, influencing future preferences and behaviours. Using the Implicit Association Test (IAT), this study aims to determine whether implicit attitudes toward specific food groups (e.g., snacks and fruits) in adulthood are shaped by childhood eating patterns. The primary hypothesis is that individuals with healthier childhood diets will show more positive implicit attitudes toward healthy foods (e.g., fruits) in adulthood than those with less-healthy childhood diets, which may show a stronger preference for unhealthy foods (e.g., snacks). This study hypothesizes that implicit rather than explicit attitudes will be stronger predictors of adult food preferences and choices. Furthermore, given the observed gender differences in food consumption, this study will also assess whether these implicit attitudes vary by gender. It expects women to demonstrate more positive implicit attitudes toward fruits and healthier eating patterns than men, who may show a stronger implicit preference for high-calorie, less-nutritious foods.

## 2. Materials and Methods

### 2.1. Participants

To determine the sample size needed to examine the associations between food preferences and implicit attitudes among healthy adults, we used a power calculation that drew effect sizes based on implicit attitudes in adults (d = 0.41) [5]. The sample size was calculated using the G*power computer software (version 3.1) based on correlation analysis, a medium effect size (d = 0.4), alpha = 0.05, and 95% power; a sample size of 67 subjects was needed. We expected that 10% of the acquired data would not be usable. Thus, we aimed to recruit more than 100 participants. Ultimately, 204 subjects were recruited for this study, with a final participation of 119 volunteers. The sample included 80 women and 39 men, all over 18 (M = 28.35 and SD = 10.5). Participants were recruited via convenience sampling, primarily from students at Tel-Hai Academic College and through social media and WhatsApp messages. Students from Tel-Hai Academic College received 0.5 credit points for their participation through the SONA system. The Tel-Hai Academic College Ethics Committee approved all procedures.

### 2.2. Measures

#### 2.2.1. Demographics

The demographic questionnaire collected data on the participant’s age, sex, religion, height, weight; childhood and current place of residence, diet status (yes/no; if yes, type of diet); vegetarian/vegan status; health status; medication use (yes/no; if yes, type of medication); and presence of psychological disorders.

#### 2.2.2. Childhood Food Preferences Questionnaire (CFPQ)

We used the CFPQ, a retrospective self-report questionnaire developed in our lab, to assess the frequency of food consumption during the participants’ childhoods [8]. The CFPQ was validated and published through meticulous procedures. Content validation involved an expert review, and a pilot test (*n* = 40) refined the questionnaire for clarity. The test-retest reliability was assessed by administering the questionnaire twice, three weeks apart, to a sample of young adults (*n* = 40), yielding a strong Pearson correlation (rp(38) = 0.78, *p* = 0.003), confirming its stability and accuracy in capturing childhood food consumption [8].

The questionnaire covers seven food categories: bread and cereals, fast food, dairy products, sweets and snacks, drinks, fruits and vegetables, and meat and fish. Each category contains various food items (e.g., pizza, fries, and hamburgers in the fast-food category). Participants rated the frequency of consumption for each item on a four-point Likert scale, ranging from 1 (“never”) to 4 (“very often”). Scores for each category were summed to represent its overall consumption in childhood. Internal consistency was assessed using Cronbach’s alpha, yielding the following results: grains (α = 0.739), fast food (α = 0.842), dairy products (α = 0.706), snacks (α = 0.901), drinks (α = 0.733), fruits and vegetables (α = 0.922), meat (α = 0.861), and overall (α = 0.814).

#### 2.2.3. Implicit Association Test (IAT)

The Implicit Association Test (IAT) aims to assess the extent to which target and reference categories have a robust mental association by measuring reaction times in a stimulus classification task. It was adapted to measure implicit attitudes toward foods [7], translated into Hebrew, and developed using the Iatgen software (version 1.2.4) [40]. This test includes two target categories— “snacks” and “fruits”—and two reference categories— “pleasant” and “unpleasant”. Each category includes five items, words semantically belonging to that category. Participants were asked to place their right and left index fingers on the keyboard’s ‘I’ and ‘E’ keys, respectively, and to complete seven blocks of the classification task, each consisting of 20 or 40 trials. In each block, different categories appeared on either side of the upper part of the screen. In each trial, an item belonging to one of the categories appeared in the centre of the screen in random order.

Participants were required to classify each item into the appropriate category by pressing the corresponding key quickly and with as few errors as possible. If a classification error occurred, a red “X” appeared on the screen, and participants were asked to correct the error by pressing the correct key. The first and second blocks were training, displaying the two target categories (Snacks and Fruits) and the reference categories (“pleasant” and “unpleasant”). The third and fourth blocks were combined blocks, in which one target category and one reference category appeared on each side of the screen (for example, on the right was “snacks” + “pleasant” and on the left was “fruits” + “unpleasant”). The fifth block was a training block that only displayed the target categories but on opposite sides to the first block to mitigate the associations learned in the previous blocks. The sixth and seventh blocks were combined blocks, where the target and reference categories appeared together but in reverse pairs compared to the third and fourth blocks. The categories’ order on the screen’s sides changed randomly between participants. Blocks 1, 2, 3, and 5 contained 20 trials each, while blocks 4 and 7 contained 40. The tool measured participants’ reaction times in classifying the items into the appropriate categories. The data analysis included the combined blocks (3, 4, 6, and 7).

This test assumes that individuals classify stimuli faster when pairings match their mental associations [40]. For instance, if someone views snacks as less pleasant than fruits, they will respond faster when “snacks” and “Unpleasant” are paired. Scores are calculated using the Iatgen software, with the data being standardized to account for individual differences in reaction times. This software calculated the intra-individual differences by dividing reaction time differences between blocks 3 and 6 and blocks 4 and 7 by their pooled standard deviations. The average of these results produced a D score for each participant. A positive D score reflects a strong association between “fruits” and “pleasant” and “snacks” and “unpleasant”, indicating positive attitudes toward fruits. A negative D score reflects the opposite. Others showed that the IAT has high reliability and validity, with higher sensitivity (IAT effect of 153.5 ms, d = 1.21) compared to Evaluative Semantic Priming (ESP effect 64.0 ms and d = 0.62) [5]. Additionally, the IAT had lower correlations with explicit attitude measures (r = 0.25) than between explicit tools (r = 0.60).

**Figure 1:**
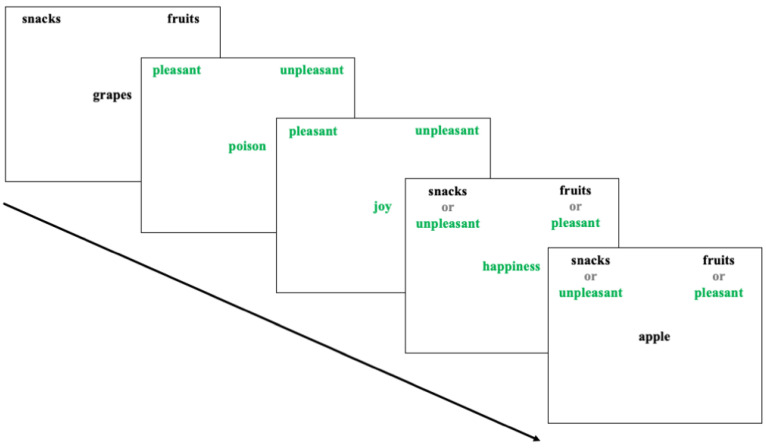
Food-related IAT.

Previous studies using food-related IATs found high internal consistency, with a Cronbach’s alpha of α = 0.80 − 0.88. Additionally, it was found that the IAT is a significant variable in the predictive model of behavioural choice (B = 0.39, SE = 0.12, and *p* = 0.001). The Iatgen software was found to have high reliability and validity in developing IATs, showing similar results to previous IATs created using other means. Reliability testing through a split-half test was high (r = 0.86). Internal consistency with Cronbach’s alpha was also high (α = 0.83 − 0.85). High convergent validity was also found, with the correlation between the tested tool and an existing tool being r(187) = 0.53, *p* < 0.001, and 95% CI [0.42, 0.63] [40]. Similar to previous tests, in the current study, the internal consistency testing using a split-half test was also found to be high (r= 0.86). (Figure 1).

### 2.3. Experimental Design

Participants completed the study using a laptop or desktop computer with a keyboard. They participated from home or another location of their choice. After signing an informed consent form, participants filled out a demographic questionnaire, followed by the Childhood Food Preferences Questionnaire (CFPQ), in which they rated the frequency of their childhood food consumption. Finally, participants completed the Implicit Association Test (IAT), which involved classifying food-related items to assess implicit attitudes. Instructions were provided before each of the seven blocks. After completing the IAT, participants saw a thank-you message.

### 2.4. Data Analysis

Descriptive statistics: for categorical variables, summary tables are provided, giving the sample size and relative frequencies, and for continuous variables, summary tables are provided, giving the arithmetic mean (M), standard deviation (SD), and range depending on the data distribution. Inferential statistics: A chi-squared was applied to test the correlations between the study group, socio-demographics, and personal characteristics. For the continuous variables related to questionnaires (subscales), Cronbach’s alpha coefficients were calculated to assess the internal consistency for each subscale of the questionnaires.

Pearson correlation coefficients were calculated to explore potential relationships between participant characteristics and food preferences. Participants were divided into quartiles based on their childhood fruit consumption to explore potential differences in implicit attitudes. An independent sample *t*-test was conducted to compare the D scores of participants in the highest and lowest quartiles of fruit consumption. The data were further stratified by gender to investigate whether the observed effects varied between men and women. Independent sample *t*-tests were conducted separately for men and women to compare D scores between high and low fruit consumption groups. A *p*-value of 5% or less was considered statistically significant. The data were analysed using SPSS version 28 (IBM).

## 3. Results

A preliminary chi-square analysis of the participants’ demographics was performed, and the results indicate a non-significant association among the variables under investigation. Therefore, the statistical analyses included no demographic variables as covariates (Table 1).

Table 2 exhibits the means and standard deviations of the childhood consumption of fruits and snacks and the “D score” found in the sample. Also, 104 subjects were found to have a positive D score, that is, to have positive latent attitudes toward fruits and negative ones toward snacks, and 15 subjects were found to have a negative D score, that is, to have positive latent attitudes toward snacks and negative ones toward fruits.

The first research hypothesis proposed a positive relationship between fruit consumption in childhood and the D score, suggesting that participants who ate more fruits as children would have more positive implicit attitudes toward fruits. The second hypothesis suggested a negative relationship between snack consumption in childhood and the D score, indicating that participants who consumed more snacks in childhood would have more positive implicit attitudes toward snacks.

To test these hypotheses, the correlations among the three variables—D score, childhood fruit consumption, and childhood snack consumption—were examined using Pearson’s correlation coefficient (see Table 3). There was no correlation between childhood fruit consumption and the participants’ D scores. Additionally, there was no correlation between childhood snack consumption and the D scores. Based on these findings, the research hypotheses were not supported. 

For further analysis, participants were divided into quartiles based on their fruit consumption during childhood. Differences between the highest and lowest quartiles were examined using an independent sample *t*-test (see Table 4). A statistically significant difference was found between the two groups, with the D scores of participants in the high-fruit-consumption quartile being higher than those in the low-consumption quartile. This indicates that participants who consumed more fruits during childhood held more positive implicit attitudes toward fruits than those with lower fruit consumption.

Additionally, the sample was divided into quartiles based on snack consumption during childhood, and differences between the highest and lowest consumption quartiles were examined using an independent sample *t*-test (see Table 5). No significant difference was found between these two groups.

To understand the differences between the high- and low-fruit-consumption groups in childhood, we examined the differences by gender. Among the women, a statistically significant difference was found between the consumption groups (see Table 6), with the D score of women with high fruit consumption in childhood being higher than that of women with low fruit consumption. This indicates that women who consumed more fruits during childhood had more positive implicit attitudes toward fruits than those with lower consumption. In contrast, no significant difference was observed among the men between the high- and low-fruit-consumption groups in childhood (see Table 7).

## 4. Discussion

The current study investigated the relationship between childhood eating habits and implicit attitudes toward food in adulthood. Contrary to the research hypotheses, no significant relationship was found between childhood fruit consumption and participants’ D scores. Similarly, no association was observed between childhood snack consumption and D scores. This suggests that childhood eating habits may not directly influence implicit attitudes toward food in adulthood. However, additional analyses revealed that participants with high childhood fruit consumption had significantly higher D scores than those with low fruit consumption. This indicates that individuals who ate more fruits as children had more positive implicit attitudes toward fruits than their counterparts. Moreover, a gender-based analysis found similar results among women. 

This study focused on a specific aspect of the Theory of Food (ToF) [8,9], namely, the relationship between childhood eating habits and implicit attitudes toward food in adulthood. This highlights how the foods consumed during childhood may be linked to the “food theory” individuals develop in adulthood. However, the ToF proposes additional variables that might influence this “food theory,” such as one’s cultural context, family and social experiences related to food, and the emotional context of food consumption [9]. Therefore, the findings of this study may not necessarily indicate a flaw in the theory but rather suggest that factors beyond childhood eating habits influenced the participants’ implicit attitudes toward food. Overall, this study explored whether childhood eating patterns, specifically, the consumption of fruits and snacks, shape implicit attitudes toward these food groups in adulthood. The results partially support this claim. The finding that higher childhood fruit consumption is associated with more positive implicit attitudes toward fruits, especially among women, suggests that early dietary habits can create lasting cognitive associations, as proposed by the ToF. However, the lack of a significant relationship between snack consumption and implicit attitudes indicates that not all childhood eating patterns may translate into implicit attitudes in adulthood. Thus, the results provide mixed support for the aim of this study. They highlight a gender-specific relationship between healthy childhood eating habits (e.g., fruit consumption) and positive implicit attitudes toward healthy foods in adulthood. This finding, along with the suggestion that the link between early consumption of less-healthy foods (e.g., snacks) and implicit attitudes may be weaker or more complex than was initially hypothesized, has significant implications for public health and nutrition education. This nuanced outcome opens the door for further exploring factors that may mediate or moderate the relationship between childhood eating habits and implicit attitudes toward food, underscoring the importance of continued research in this area.

Indeed, the literature provides evidence of other variables affecting implicit attitudes toward food. For instance, implicit attitudes can be influenced by an individual’s knowledge about the healthiness of foods, where exposure to images of health risks associated with unhealthy foods affects participants’ implicit attitudes toward those foods [41]. Additionally, values and beliefs also play a role, with vegan participants exhibiting more negative implicit attitudes toward animal-based foods than vegetarians and vegetarians demonstrating more negative attitudes than omnivores. Other factors, such as one’s moral views on food and personality traits like empathy, have also been shown to affect implicit food attitudes [42].

Furthermore, ongoing dietary restrictions influence implicit attitudes toward food. Individuals on long-term restrictive diets tend to display less -positive implicit attitudes toward tasty foods than control groups [43]. Additionally, childhood food-related memories, such as being rewarded with food or having a controlled diet, have been linked to the higher consumption of sweet and salty snacks in adulthood. On the other hand, guidance toward healthy eating and restrictions were associated with higher consumption of fruits and vegetables [44]. Given that implicit attitudes are connected to eating habits and dietary choices [7], it can be inferred that childhood eating memories influence food consumption habits and implicit attitudes in adulthood.

Another potential explanation for the lack of association between childhood eating habits and implicit attitudes in this study may be related to the validity of the ToF. The findings of this study can be seen as a partial challenge to this theory, suggesting that further research is needed to understand better the complex relationships between childhood dietary habits and various cognitive and emotional variables. When the sample was divided into quartiles reflecting low versus high fruit consumption, differences emerged in D scores, with the high-consumption group displaying higher D scores. This finding suggests a trend toward the research hypothesis, and increasing the sample size in future studies could reveal the anticipated effect in correlation and difference analyses.

To further explore these differences, the sample was analysed by gender. Among the women, those with high childhood fruit consumption had significantly higher D scores than those with low fruit consumption. This difference was not observed among the men. However, it is important to interpret this result cautiously, given the small sample size, particularly in the quartile analysis, where each group contained fewer than 30 participants. This may lead to potential issues with the normality assumption required for statistical tests. 

Nonetheless, the more positive implicit attitudes of women in the high-fruit-consumption group could be influenced by additional variables. For example, a study found that women who enjoyed shopping for and preparing food consumed more fruits and vegetables than those who did not enjoy these activities [45]. This suggests that such behaviours influence women’s implicit attitudes and explain the observed differences.

Previous research has also indicated gender differences in food-related attitudes, with women being reported to consume more fruits, avoid high-fat foods, and limit salty food intake compared to men [46]. Women tend to place greater importance on the healthiness of foods, while men often prioritize taste [47]. Additionally, women generally know more about the health effects of their food than men [48]. These attitude differences are likely reflected at the implicit level, potentially accounting for the gender differences observed in this study. Other research has shown that women’s preferences for healthy food are primarily driven by more excellent nutritional knowledge and a higher motivation to maintain weight than men [49], further supporting the bias in women’s implicit attitudes toward healthier foods.

An important aspect to consider in understanding childhood eating behaviours and their lasting effects into adulthood is the role of the household’s financial and human capital. Numerous studies have shown that children from affluent households typically have greater access to nutritious foods, face fewer financial barriers, and are more likely to maintain healthier dietary habits into adulthood [50,51,52]. These advantages are often reinforced by parental attitudes toward child-rearing, particularly nutrition. Parents with higher educational backgrounds or human capital tend to prioritize the availability of healthy food options, serve as role models for balanced eating, and engage in positive feeding practices that influence their children’s long-term dietary patterns [50,53]. Additionally, children from households of a higher socioeconomic status (SES) often have greater food autonomy and face fewer constraints regarding food choices in adulthood, reinforcing positive food behaviours and shaping their implicit attitudes toward certain foods [54,55]. Given that the current study identified a positive relationship between higher childhood fruit consumption and favourable implicit attitudes toward fruits, it is plausible that socioeconomic and familial factors shaped these observed attitudes. Future research should explore how one’s SES, parental attitudes, and access to food during childhood influence their implicit food attitudes in adulthood.

The current study’s findings may also be influenced by limitations related to the tools used. One primary limitation concerns the validity and reliability of the Implicit Association Test (IAT). Some have identified issues with the generalizability and reproducibility of the IAT [56]. In contrast, others have noted its low test–retest reliability [57], potentially due to the reliance on reaction times, where even a tenth of a second can significantly affect scores. Additionally, the IAT’s relative nature complicates interpretation, as a strong association between “fruits” and “pleasant” may also reflect a solid opposite association between “snacks” and “unpleasant” [58]. Another limitation is the reliance on recall questionnaires for childhood eating habits, which may have led to inaccuracies due to the passage of time [59,60,61]. The sample characteristics may also limit the generalizability of the findings, as the average D score was notably higher than that found in previous research [7], with fewer participants showing negative D scores, suggesting a tendency toward positive implicit attitudes toward fruits and negative attitudes toward snacks.

While this study did not fully validate the ToF, it provides avenues for further research into additional variables that may influence implicit food attitudes. Future studies should explore these factors and employ different methodologies, such as longitudinal studies, to assess childhood dietary habits and their impact on adult implicit attitudes. Psycho-physiological measures (e.g., EEGs, skin conductance, and heart rates) could also be used to evaluate participants’ responses to various foods in adulthood. Additionally, increasing the sample size could help clarify the differences between the high and low-fruit consumption groups by allowing subgroup analyses based on gender and dietary preferences (e.g., vegan or vegetarian).

## 5. Recommendations for Policy and Practice in Promoting Healthy Eating Habits

Based on this study’s findings, policymakers are encouraged to implement several early intervention strategies to promote public health. Educational programs aimed at parents and caregivers should emphasize the importance of establishing healthy eating habits from a young age, particularly regarding fruit consumption, highlighting their positive impact on children’s implicit attitudes and lifelong behaviours. Additionally, initiatives that increase access to healthy foods in low-income neighbourhoods, such as subsidizing fruits and vegetables in schools and community programs, can improve children’s dietary habits. Incorporating nutrition education into school curricula will further reinforce healthy eating habits by providing children with knowledge about the benefits of fruits and vegetables. Lastly, ongoing research should explore how childhood eating habits influence adult food preferences, mainly focusing on gender differences and socioeconomic factors to inform tailored interventions.

## 6. Conclusions

In conclusion, while the current study did not find a direct relationship between childhood eating habits and implicit attitudes toward food in adulthood, it did reveal that individuals with higher childhood fruit consumption exhibited more positive implicit attitudes toward fruits. These results, particularly among women, suggest that childhood experiences with certain foods may contribute to implicit preferences later in life. However, these findings are limited by the study’s sample size and methodological constraints. The study’s results also underscore the complexity of food-related attitudes, as other factors, such as gender, dietary preferences, and broader cultural influences, likely play a significant role. Future research should aim to investigate these variables more thoroughly, utilizing more extensive and diverse samples, longitudinal designs, and alternative measures to capture the nuanced relationship between early dietary habits and implicit food attitudes in adulthood.

## Figures and Tables

**Table 1:** The demographics and characteristics of the study’s sample.

	Mean	STDEV	Range		
Age	28.35	10.50	19–81		
	**Males**	**Females**			
Gender	36 (32.77%)	80 (67.23%)			
	**City**	**Kibbutz**	**Cooperative Settlement**	**Other**	
Current living place	58 (48.74%)	30 (25.21%)	14 (11.76%)	17 (14.29%)	
Childhood living place	63 (52.94%)	11 (9.24%)	15 (12.61%)	30 (25.21%)	
	**Muslim**	**Christian**	**Jew**	**Druze**	**Other**
Religion	5 (4.2%)	2 (1.68%)	104 (87.39%)	3 (2.52%)	5 (4.21%)
	**Yes**	**No**			
Health Diet	26 (21.85%)	93 (78.15%)			
	**Excellent**	**Good**	**Reasonable**	**Not so Good**	**Bad**
Health Status	49 (41.18%)	58 (48.74%)	9 (7.56%)	2 (1.68%)	1 (0.84%)
	**Carnivore**	**Vegetarian**	**Vegan**		
Diet Preference	73 (61.34%)	32 (26.89%)	14 (11.77%)		

**Table 2:** Means and standard deviations of childhood fruit and snack consumption and “D score”.

Variable	Mean	SD
Childhood Fruit Consumption	3.01	0.765
Childhood Snack Consumption	2.519	0.705
D Score	0.504	0.443

**Table 3:** Pearson correlations between the variables “D score”, “childhood fruit consumption”, and “childhood snack consumption” (N = 119).

		M	SD	1		2	
				*r*	*p* Value	*r*	*p* Value
1	D Score	0.50	0.44	---			
2	Childhood fruit consumption	3.01	0.76	0.118	0.201	---	
3	Childhood snack consumption	2.51	0.70	0.000	0.996	0.335	<0.001

**Table 4:** Differences in D scores between the groups “low fruit consumption in childhood” and “high fruit consumption in childhood”.

	Low Childhood Fruit Consumption (n = 32)		High Childhood Fruit Consumption (n = 29)				
	M	SD	M	SD	t(59)	*p* Value	Cohen’s d
D Score	0.36	0.56	0.57	0.37	−1.67	0.005	0.44

**Table 5:** Differences in D scores between the groups “low snack consumption in childhood” and “high snack consumption in childhood”.

	Low Childhood Snack Consumption (n = 34)		High Childhood Snack Consumption (n = 25)				
	M	SD	M	SD	t(57)	*p* Value	Cohen’s d
D Score	0.58	0.43	0.48	0.41	0.81	0.20	0.23

**Table 6:** Differences in D scores between the groups “low fruit consumption in childhood” and “high fruit consumption in childhood” among women.

	Low Childhood Fruit Consumption (n = 18)		High Childhood Fruit Consumption (n = 20)				
	M	SD	M	SD	t(36)	*p* Value	Cohen’s d
D Score	0.030	0.057	0.58	0.042	−1.72	0.040	0.55

**Table 7:** Differences in D scores between the groups “low fruit consumption in childhood” and “high fruit consumption in childhood” among men.

	Low Childhood Fruit Consumption (n = 14)		High Childhood Fruit Consumption (n = 9)				
	M	SD	M	SD	t(36)	*p* Value	Cohen’s d
D Score	0.044	0.055	0.054	0.026	−0.51	0.30	0.23

## Data Availability

The datasets generated and/or analysed during the current study are not publicly available due to their containing information that could compromise the privacy of research participants but are available from the corresponding author upon reasonable request.
